# Chemical, Physical, and Sensory Effects of the Use of Bentonite at Different Stages of the Production of Traditional Sparkling Wines

**DOI:** 10.3390/foods10020390

**Published:** 2021-02-10

**Authors:** Cristina Ubeda, María Ignacia Lambert-Royo, Mariona Gil i Cortiella, Rubén Del Barrio-Galán, Álvaro Peña-Neira

**Affiliations:** 1 Departamento de Nutrición y Bromatología, Toxicología y Medicina Legal, Facultad de Farmacia, Universidad de Sevilla, C/Profesor García González 2, 41012 Sevilla, Spain; 2 Instituto de Ciencias Biomédicas, Facultad de Ciencias, Universidad Autónoma de Chile, Santiago 8910060, Chile; 3 Facultad de Ciencias Agronómicas, Universidad de Chile, Avenida Santa Rosa 11315, Santiago 8820808, Chile; milambertr@hotmail.com (M.I.L.-R.); rdelbarriogalan@gmail.com (R.D.B.-G.); apena@uchile.cl (Á.P.-N.); 4 Instituto de Ciencias Químicas Aplicadas, Facultad de Ingeniería, Universidad Autónoma de Chile, Santiago 8910060, Chile; mariona.gil@uautonoma.cl

**Keywords:** sparkling wines, bentonite, volatile compounds, foam properties, sensory analysis

## Abstract

The addition of bentonite to wine to eliminate unstable haze-forming proteins and as a riddling adjuvant in the *remuage* is not selective, and other important molecules are lost in this process. The moment of the addition of bentonite is a key factor. Volatile profile (SPME-GC-MS), foam characteristics (Mosalux method), and sensory analyses were performed to study the effect of the distribution of the dosage of bentonite for stabilization of the wine among the addition on the base wine before the *tirage* (50%, 75%, and 100% bentonite dosage) and during the *tirage* (addition of the remaining dosage for each case). Results showed that the addition of 50% of the bentonite to the base wine (before the *tirage*) resulted in sparkling wines with the lowest quantity of volatile compounds, mainly esters and norisoprenoids. No significant differences were found among the sparkling wines after 9 months of aging in relation to foam properties measured by Mosalux, although higher foamability and crown’s persistence were perceived in the sparkling wines with the addition of 75% and 100% of the bentonite dosage in sensory trials. The results of this study suggested that the amount of bentonite added as a fining agent in the *tirage* had greater effects than during the addition of this agent in the base wine.

## 1. Introduction

The refusal of a wine by consumers can be driven by several reasons, some of which are subjective and others objective, such as defects in the product. Currently, haze formation in wines is an important concern for the industry because turbidity is one of the main causes of faulty perception by consumers and huge monetary losses due to the direct decrease of the quality of the wines and to the wine loss [1,2].

Different types of molecules such as polysaccharides, polyphenols, or proteins can contribute to the formation of haze owing to their instability [2]. Among them, proteins, and more specifically, proteins from grapes, seem to be the major contributors to the formation of haze [3]. The most employed strategy for the elimination of these unstable proteins is to treat wines with bentonite before bottling them.

Bentonite is a natural clay mineral with a high amount of montmorillonite, which is negatively charged at wine pH and interacts electrostatically with the positively charged wine proteins that are adsorbed on the bentonite surface, thus producing flocculation, and removing them from the wine [4].

During sparkling wine production, bentonite is generally used twice during the production process. First, it is added as a fining agent to achieve protein stabilization of the base wine, preventing the future formation of protein haze. Second, a small amount of bentonite is added as an adjuvant to the tirage solution to facilitate the flocculation of yeast strains during the process of riddling [5]. The lack of an available alternative for bentonite during sparkling wine production at the industrial scale, its high effectiveness, its simple application procedure, and its low cost explain its widespread use in wineries despite its negative effect on the foam quality and aromatic profile of wines [6,7,8,9]. Bentonite does not bind selectively to unstable proteins; thus, it also removes other positively charged species or aggregates. The loss of volatile compounds can occur directly, via the adsorption of these compounds onto bentonite [10], or indirectly, when the aromas are fixed by proteins or polysaccharides; moreover, some of the aromas are also discarded after the elimination of bentonite along with these macromolecules [6,7,8,11,12].

Furthermore, protein removal itself has its drawbacks, because the proteins and polysaccharides removed by bentonite affect the foamability of a sparkling wine [13,14]. Specifically, the addition of bentonite as a fining agent decreases foamability [15]; moreover, when added to facilitate the riddling process, bentonite significantly affects the foam quality, decreasing the maximum height and persistence of the wine foam [9,16].

Among the main types of proteins found in sparkling wine, i.e., those from grapes (chitinases and thaumatin-like proteins) and yeast (mainly mannoproteins), the ones from the fruit seem to play a major role in protein haze formation [17]. To overcome the disadvantages of the employment of bentonite, new additives are being sought to compensate for foam depreciation [18,19].

Despite all these negative effects that it has on the final product, a specific amount of bentonite is required to achieve protein stabilization of base wines, and it is still the most widely employed and effective agent in wine protein stabilization [20]. Hence, defining the appropriate application dosage of bentonite is extremely important for using enough to prevent haze. However, applying an excess of bentonite is not recommended, firstly to ensure the nitrogen quantity needed for fermentation (if it is added to the must or for the second fermentation) and secondly, as mentioned above, to prevent negative sensorial effects. It has been described that “matrix factors” modulating the removal of wine odor-active compounds during bentonite fining are the chemical nature of the clay, the hydrophobicity, the initial concentration of wine odor-active compounds, and the abundance and nature of wine proteins [7,10,12]. In this sense, ethyl esters seem to be the most affected volatiles, significantly decreasing their presence after bentonite treatment [7,10,12].

In addition to the quantity added, the moment of its addition is key to preserving the sensory characteristics of the wine. Some researchers have investigated the implications of bentonite treatments at different time points of the production process, especially before, during, and after fermentation [4,8,17,21]. Thus, Lambri et al. [8] concluded that a smaller dose of fining agent is needed when bentonite is added only to the must. However, other authors observed that the addition of bentonite during fermentation minimized both the dose amount required to allow wine protein stabilization and the negative sensory implications [4,22]. These results were in agreement with the ones of Lira et al. [17], who established that the best moment of addition of bentonite in Albariño wines was during alcoholic fermentation, particularly at the middle and at the end, giving rise to wines with higher aromatic intensity, being also preferred by the consumers in their sensory trials. Moreover, the application of bentonite at the middle and end of fermentation seems to provide better foaming properties to the wine [21].

However, despite the results found in these studies, the effects of the distribution of the needed dosage for stabilization during different phases of sparkling wine production have not been studied.

The aim of this study was to determine such effects on two essential quality parameters of sparkling wines: volatile composition and foam properties. A Chardonnay sparkling wine was tested, and the protein stabilization dosage of bentonite was distributed among the stages of fining of the base wine and before the second fermentation of the tirage liquor (as a riddling adjuvant) in different proportions. In addition, sensory analysis was conducted to corroborate and establish the effects/implications of a higher or lower bentonite dosage added at each stage.

## 2. Materials and Methods

### 2.1. Winemaking and Experimental Design

The study was carried out using a Chardonnay base wine variety of the 2017 vintage made in the San Pedro de Tarapacá winery, which is located in the Casablanca Valley region of Chile. This base wine had a 10.4% vol. and a pH of 3.42.

**Figure 1:**
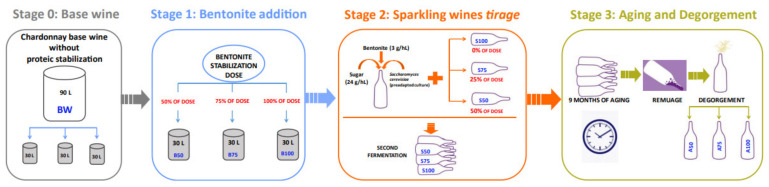
Schedule of the production process and the samples analyzed.

The stabilization dosage of bentonite for this base wine, which was determined using a fast heat test, was 17 g/hL [23]. For this study, activated sodium bentonite was used (SIHA^®^ G, Eaton Industries, Dublin, Ireland). Figure 1 shows a detailed scheme of the experiment and production process of the sparkling wines of this study. Ninety liters of the base wine were distributed into three stainless steel tanks (STAGE 0) (Figure 1). Each tank was treated with 50%, 75%, and 100% of the stabilization dosage, i.e., 8.5, 12.75, and 17 g/hL, respectively. Bentonite was added as a 5% bentonite solution in water, and it was hydrated with cold water 24 h before the application. Bentonite acted for 72 h, after which the wine of every tank was racked off and transferred to clean stainless-steel tanks (B50, B75, and B100) (STAGE 1) (Figure 1). Following the traditional method (champenoise), the tirage was carried out. A preadapted yeast culture of Lalvin EC1118^®^ *Saccharomyces cerevisiae bayanus* purchased from Lallemand (Chile) was used for a second fermentation in the bottle (750 mL green bottle Maipo type, Cristalchile, Chile). For the preadaptation of the yeast, 40 g of yeast were dissolved in 400 mL of water at 35 °C. After 30 min, this mixture was added to 1 L of water containing 200 g of sugar perfectly dissolved, and following this, 4.5 L of wine were incorporated slowly. This mixture was incubated overnight at 25–30 °C. Next day, a viable yeast cell counting, and density measuring were done. The addition of sugar, water, and base wine was repeated but gradually increasing the base wine volume until the *tirage* to force the yeast to adapt to the rough conditions of this matrix. At this point, a second addition of bentonite was carried out, adding to every bottle of base wine 24 g/L of sucrose, preadapted yeast, and the bentonite necessary for each treatment. Hence, the bottles of the base wines for the treatments, 50% and 75% (S50 and S75) were spiked with the dosage of hydrated 5% (m/v) bentonite needed to complete their stabilization; this was 8.5 and 4.25 g/hL, respectively. Moreover, 3 g/hL bentonite was added to all the bottles to facilitate riddling, avoid differences due to technological reasons, and to be able to assign the results and effects to the bentonite used as a clarifying agent. Fifteen days later, the second fermentation was complete, and samples were taken (S50, S75, and S100) (STAGE 2) (Figure 1). After 9 months of aging on lees at 16 °C, the remuage was carried out in one cycle with a Gyropalette^®^ (Oenoconcept^®^, Epernay, Champagne, France), and sparkling wines were finished (A50, A75, and A100) (STAGE 3) (Figure 1). The resulting sparkling wines presented an alcoholic degree of 11.9 ± 0.1 and a pH of 3.31 ± 0.01. Stage 0 and Stage 1 wines were analyzed in triplicates (analytical replicates), and three bottles each of the wines from Stage 2 and Stage 3 were analyzed at each condition (biological replicates).

### 2.2. Reagents and Standards

The standard compounds employed in this study for the identification and quantification, i.e., ethyl butyrate, isoamyl acetate, ethyl hexanoate, hexyl acetate, ethyl lactate, ethyl octanoate, isoamyl hexanoate, ethyl nonanoate, methyl decanoate, ethyl decanoate, isoamyl octanoate, diethyl succinate, β-phenethyl acetate, isoamyl decanoate, isobutanol, isoamyl alcohol, hexanol, E-3-hexenol, phenylethyl alcohol, linalool, α-terpineol, citronellol, E-nerolidol, hexanoic acid, octanoic acid, and decanoic acid, were supplied by Sigma-Aldrich (Germany). Sodium chloride and 4-methyl-2-pentanol (internal standard) were purchased from Merck (Darmstadt, Germany).

### 2.3. Volatile Compound Analysis

Volatile compounds were extracted using headspace solid phase microextraction (HS-SPME), as described by Ubeda et al. [24]. For the extraction, a 2 cm 50/30 μm fiber made of carboxen/divinylbenzene/polydimethylsiloxane (Supelco, Bellefonte, PA, USA) was employed. For the identification of compounds, authentic reference standards were used, and matching was done with the 2.0 version of the standard NIST library and the linear retention indices (LRIs) from the literature (Pherobase; www.pherobase.com) and the NIST Mass Spectrometry Data Center; https://webbook.nist.gov/ (accessed on 20 November 2020)). LRIs were calculated using the retention times of *n*-alkanes (C6–C30) under identical conditions for each analysis temperature program. All data were expressed as concentrations (μg/L) obtained from calibration curves using the reference standards (relative area vs. concentration), except in the case of C13 norisoprenoids, for which the data were expressed as relative areas. The relative area was calculated by dividing the peak area of the major ion of each compound by the peak area of the major ion of the internal standard.

### 2.4. Determination of Foaming Properties

Foam properties were measured using the Mosalux procedure [25,26]. To carry out the measurement, the wines were degassed. Thereafter, a test tube with a porous piece of glass at the bottom and a CO_2_ entry was filled with 100 mL of the sample, and a constant flow of CO_2_ (10 L/h) was passed through the sample at a constant temperature of 16 °C. The parameters measured were HM, which is the maximum height reached by the foam and represents the foamability, and HS, which is the stable height of the foam that represents the ability of the wine to produce stable foam/persistence of the foam collar [25]. These analyses were performed in triplicates. The parameters, HM and HS, were expressed in millimeters.

### 2.5. Sensorial Analyses

Samples of sparkling wines after 9 months of aging were employed for sensory analysis: A50, A75, and A100 (addition of 50%, 75%, and 100% of the required dose of bentonite, respectively, to the base wines). They were evaluated by an expert panel of 17 tasters who are professional oenologists from the sparkling wine industry in Chile (six females and eleven males). The attributes selected were aromatic intensity, foamability, foam stability, and CO_2_ integration. The last attribute provides information about foam aggressiveness in the mouth. For each evaluation, 50 mL of sparkling wine at 8 °C was served in each glass (Riedel^®^, Riedel Crystal America Inc. Kufstein, Austria). The selected attributes were indicated on a tasting card, and panelists were asked to rank each descriptor on a 15 cm unstructured scale (from unnoticeable to very strong).

### 2.6. Statistics

The InfoStat 2017p software (Free software. FCA-Universidad Nacional de Córdoba, Argentina. www.infostat.com.ar) was used for data analysis. The means were compared using ANOVA and a post hoc (Tukey test) (α = 0.05). Principal component analysis (PCA) was performed using IBM SPSS Statistics 26 software (IBM, Barcelona, Spain). Sensory analysis data were processed using PanelCheck V1.4.2 (Free software, Norway. www.panelcheck.com).

## 3. Results and Discussion

### 3.1. Effects on Volatile Compound Profile

**Figure 2:**
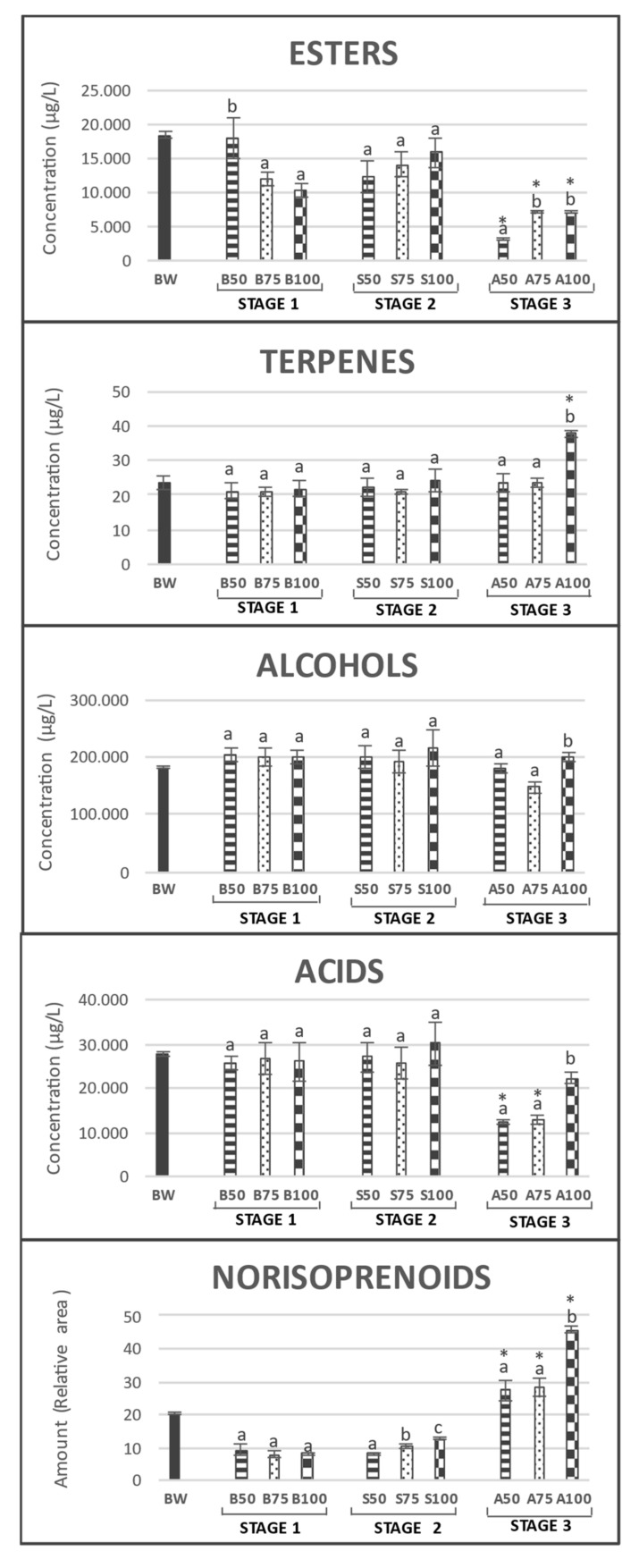
Total amounts of esters, alcohols, terpenes, acids, and norisoprenoids present in every Scheme 50. B75, B100 (wines before second fermentation with the first addition of bentonite); S50, S75, S100 (sparkling wines after second fermentation with the second addition of bentonite); A50, A75, A100 (sparkling wines after 9 months of aging on lees). Bars with different superscript letters indicate statistically significant differences (*p* < 0.05) by Tukey test among the samples belonging to the same stage *: statistically significant differences (*p* < 0.05) by Tukey test with the same treatment of the previous stage.

It has been demonstrated that the addition of bentonite to wine for fining purposes provokes an indirect removal of most of the fermentative aromatic compounds linked to the proteins removed, and only a few odor-active molecules are directly removed through adsorption [6,7,27]. Every chemical group studied (esters, alcohols, acids, terpenes, and norisoprenoids) among the 35 volatile compounds determined showed different tendencies; however, the most affected group by the bentonite treatment applied was the esters. The trend observed after the first addition of 75% (B75) and 100% (B100) of bentonite dosage seems to have caused the highest decrease in ester contents with respect to the base wines; however, the trend of these compounds after the addition of 50% of bentonite dosage (B50) was similar with respect to the base wine (Figure 2). The main compounds responsible for the strong decrease in ester contents after the addition of bentonite were ethyl butyrate, isoamyl acetate, and ethyl hexanoate (Table 1). These molecules are hydrophobic and easily adsorb on the clay of the fining agent [10]. This result agrees with that of Lambri et al. [7], who reported that the most affected esters after the application of bentonite to a white wine were ethyl butyrate, hexanoate, octanoate, isoamyl acetate, and phenylethyl acetate. In addition, hexyl acetatecontents decreased dramatically (between 73% and 82%) after the addition of bentonite in the three treatments. In contrast with these results, it was observed that stage 1 base wines presented an increase of ethyl octanoate and decanoate with respect to the stage 0 base wine (Table 1). This agrees with the results of Pozo-Bayón et al. [28], who also observed that the main changes affected ethyl octanoate and ethyl decanoate while testing the addition of bentonite in the tirage solution vs. non-addition. In contrast to the results found by Vincenzi et al. [12], we did not observe a correlation between the length of the hydrocarbon chain and the decrease in volatile compound contents.

The second fermentation and second addition of bentonite gave rise to sparkling wines that were not equally affected (stage 2). In the case of the addition of 50% of the dosage of bentonite in the tirage (S50), the resulting sparkling wines presented a significantly lower ester amount than B50 wines; however, S75 and S100 revealed an increase in the total amount of these compounds, which was probably due to the esters formed during the second fermentation. The increase in the ethyl butyrate and isoamyl acetate contents of S75 and S100 was statistically significant (Table 1). The loss of these two compounds (ethyl butyrate and isoamyl acetate) after the first addition and the increase after the second addition of bentonite could be explained by observing the macromolecular colloids present in the wine. In the first addition, i.e., a base wine without stabilization, proteins from grapes were present, and in the second addition, proteins from the yeast material involved in the second fermentation were present. Therefore, as suggested by Lambri et al. [7], these compounds may be easily attracted by proteins from the grape being mostly removed from the wine, and after the addition of bentonite in the tirage solution, they have more affinity for proteins from the yeast material, as they are not removed during the disgorgement.

Finally, as expected, the aging on lees produced a significant decrease in the total amount of esters (Stage 3), which was probably due to acid hydrolysis or even adsorption on the lees [29,30,31]. Although this was a massive loss of the ester content, the sparkling wines A75 and A100 preserved these volatile compounds more successfully than A50 (Figure 2). Isoamyl acetate, ethyl hexanoate, and ethyl octanoate were the compounds mainly responsible for this significant difference, because they were better preserved during the aging period in A75 and A100 (Table 1). This indicates that these compounds are easily bound to bentonite or the proteins that are removed with the fining agent, because their contents decreased dramatically after the first addition of bentonite and again when a high dosage of the fining agent was added to the tirage solution.

Unexpectedly, the amounts of ethyl lactate and isoamyl decanoate increased in wine A50 from the end of the second fermentation until the end of 9 months of bottle aging with lees.

Neither the first nor the second addition of bentonite produced significant differences in the total amount of alcohols in base wines at stage 1 (B50, B75, and B100) or sparkling wines at stage 2 (S50, S75, and S100) (Figure 2). Likewise, the second fermentation process did not give rise to significant differences among the total alcohols present in sparkling wines with respect to the base wines from which they were prepared. However, after 9 months of aging on lees (stage 3), the total amount of alcohols decreased significantly from stage 2 in A100 sparkling wines. The C6 alcohols, hexanol and E-3-hexenol, did not experience significant changes due to the first addition of bentonite, contrary to the results of Lambri et al. [7] but in accordance with those of Horvat et al. [22]. Nevertheless, the second addition plus the aging time decreased the contents of alcohols; however, this was not significant in almost all cases.

The terpenes group presented the same behavior as alcohols, without significant changes between the different treatments after the first and second addition of bentonite and between stages 1 and 2. The significant changes occurred after the aging period; the linalool content decreased and the citronellol content increased in all the sparkling wines studied, whereas the nerolidol content increased in A100. It is expected that during aging on lees, the effect of β-glucosidase enzyme activity releases the aglycone (odoriferous molecule) from the sugar in the volatile compounds present in their glycosidic form in the wine, thereby increasing their presence in the matrix during aging [32]. However, enzymes present in the wine matrix may disappear due to the addition of bentonite [21,33]. Hence, the treatments in which a higher dosage of bentonite was added to the tirage solution probably had less β-glucosidase enzyme available to act in the matrix because of its affinity to bentonite. Therefore, only A100, which had 3 g/hL of bentonite added as a riddling adjuvant, presented an increase in the contents of these compounds.

Similar to alcohols and terpenes, acids showed no significant differences between stages 1 and 2 and among the different treatments. Again, a decrease in the contents of all the acids determined in A50 and A75 was observed only after the aging period. In the case of A100, only the decanoic acid content significantly diminished (Table 1).

It is well known that some norisoprenoids such as 1,1,6-Trimethyl-1,2-dihydronaphthalene (TDN)and vitispiranes are aging markers [31,34]. These compounds tend to increase in content with aging time. Therefore, no significant differences were observed between stages 1 and 2; however, between stages 2 and 3 (end of second fermentation and after 9 months on lees), there was a significant increase in their amounts (Figure 2). The first addition of bentonite did not make any difference in the three treatments, but the addition in the tirage solution produced remarkable dissimilarities. The wines with the lower dosage added in this step, S75 and S100, presented slightly higher amounts of vitispirane A and TDN than S50. Although, after 9 months on lees, that difference reduced even more, and only the TDN content in A100 sparkling wines was significantly higher than that in A50 and A75.

The diversity of the effects experienced by the different volatile compounds after bentonite treatment may be explained by the fact that only a few odor-active compounds are directly adsorbed by bentonite, most of which are removed as an indirect effect of deproteinization [7]. Depending on the hydrophilic or hydrophobic characteristics of the volatile compounds, they are linked to the surface of the proteins through weak hydrogen bonds or to interior protein sites, respectively [7,12].

In general, the most effective treatment was the application of a 100% dosage of bentonite to the base wine before the second fermentation. These results agree with those obtained by several authors, indicating that bentonite fining could have a lower impact on the aroma quality when used before fermentation, i.e., when the fermentative aroma is yet to be produced [12].

### 3.2. Impact on Foaming Properties

**Figure 3:**
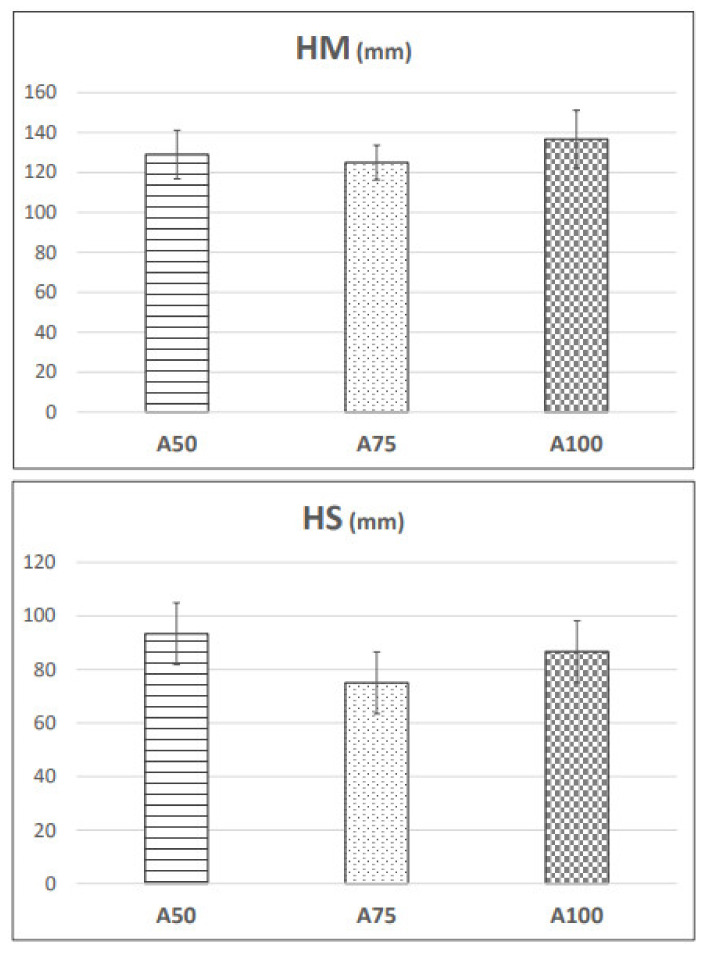
Foam properties of sparkling wines after 9 months of aging on lees from the STAGE 3 (A50, A75, A100) measured by the Mosalux method. HM: Foam maximum height; HS: Foam stability height.

Foaming properties of sparkling wines after 9 months on lees (A50, A75, and A100) were measured using the Mosalux methodology. Measurements taken using this methodology are quite heterogeneous; in this study, one measurement was taken for each bottle of the triplicates (biological replicates). Therefore, heterogenicity did not allow us to obtain significant differences among the different treatments (Figure 3). Instead, the results reflected a slightly non-significant higher maximum foam height in A100 than in A50 and A75 (Figure 3). All the sparkling wines analyzed received the same dosage of bentonite during the process but in two different stages of production. The addition of bentonite supposes the loss, among other molecules, of proteins from grapes and mannoproteins from yeast, which, as mentioned previously, are greatly responsible for sparkling wine foaming. Our results suggest that the molecules removed before the tirage are less responsible for the HM of the foam than the compounds released into the wine during aging. Previous studies have reported that glycoproteins, especially yeast mannoproteins, rather than grape proteins, more significantly affect the foaming properties of sparkling wines [35]. Reconstitution experiments performed by adding different molecular fractions isolated from wine to a model solution have pointed out the key role of mannoproteins in determining the capacity and stability of foam [14,36]. It has been reported that the glycosylated protein removal rate with sodium bentonite is low, as observed by Jaeckels et al. [37]. However, despite this, our results showed that the massive removal of thaumatin-like proteins (which play a major role in haze formation and the turbidity potential) from grapes in stage 1 after the addition of bentonite in the base wine seemed to affect the foam maximum height to a lesser extent than yeast proteins removed due to the addition of bentonite in stage 2. However, despite the absence of statistical significance, the HS results reflected that A50 showed slightly non-significant higher foam stability than A75 and A100 (Figure 3). This was not expected, since Kupfer et al. [38] described the key role of the yeast protein PAU5 in foam stability, showing that most of its removal occurred when bentonite fining of the wine was conducted before bottling. It might be that some compounds with foam stability properties from grapes are being removed; however, much research needs to be done to determine the effects of the stabilization of proteins from grapes.

### 3.3. Sensory Effects

**Figure 4:**
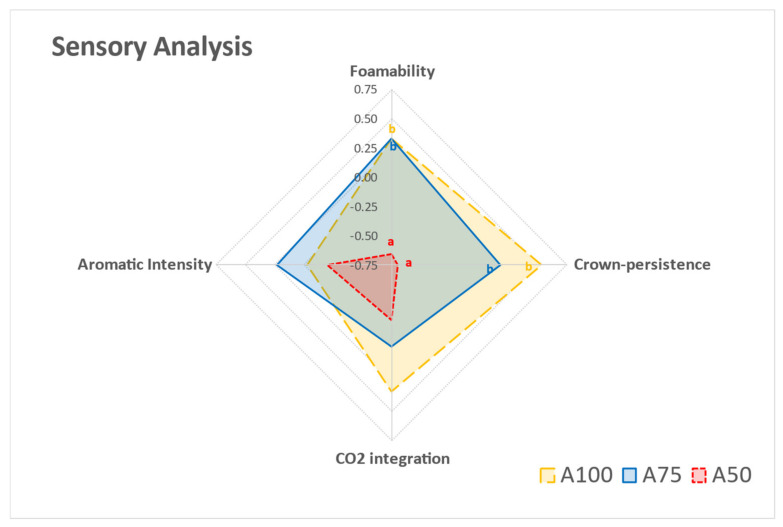
Sensory analysis of the sparkling wines after 9 months of aging on lees from STAGE 3 (A50, A75, A100). Different superscript letters in a sensorial attribute indicate statistically significant differences (*p* < 0.05) among the samples.

A simple descriptive sensory analysis was performed with sparkling wines to assess whether their chemical and physical properties were perceived. Visual parameters were strongly influenced by the distribution of the bentonite dosages during the production process. Hence, A75 and A100 showed significantly higher foamability and persistence of the crown than A50 (Figure 4). The perceived foamability agreed with the non-significant Mosalux results, whereas the persistence did not. Sensory analysis reflected the expected results, which was probably due to the lack of significance of the Mosalux results, owing to the heterogeneity of the measurements. Aromatic intensity did not show significant differences among treatments. However, A75 was perceived to be more intense, followed by A100 and A50, which was probably because of the presence of a significantly lower quantity of isobutanol and isoamyl alcohol (Table 1, Figure 4). Higher quantities of these alcohols in red wine have been previously reported as blockers of the perception of fruity attributes [39,40]. It is possible that the lower concentrations of these alcohols in A75 allowed the perception of other nuances in the wine as more intense. Martínez-Rodríguez and Polo [41] observed that the addition of 3 g/100 L of sodium bentonite to the tirage solution increased the aroma intensity and quality of sparkling wines, unlike not adding bentonite to the tirage solution at all. Perhaps the addition of 25% of bentonite dosage to the base wine in the tirage step enhanced the intensity. However, as the dosage increased, less intensity was perceived for the convergence of the higher alcohol prevalence and the yeast protein-trapping effect.

Finally, the samples did not present significant differences in in-mouth CO_2_ integration; however, it seemed that as the quantity of bentonite added prior to aging time increased, the integration of CO_2_ perceived by the panelists decreased. This parameter affects the dissolved carbon dioxide in the wine and directly influences the frequency of bubble formation in the glass, the growth rate of rising bubbles, the mouthfeel, and the aromatic perception [42].

### 3.4. Multivariate Analysis

**Figure 5:**
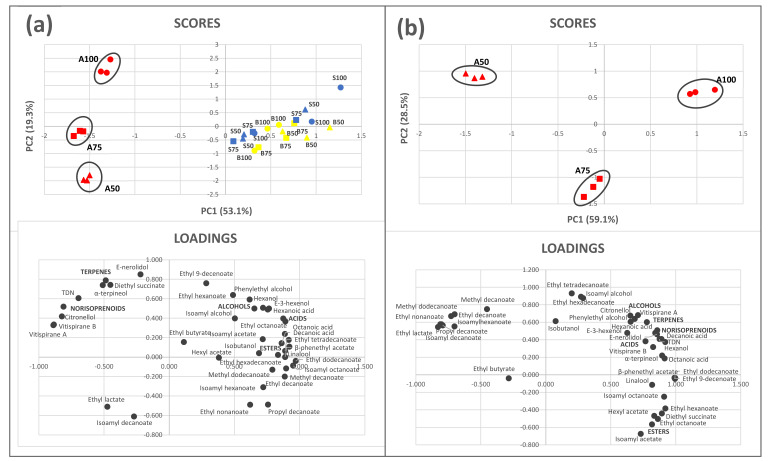
Data scores and loading biplot on the plane of the first two principal components (PC1 against PC2) of (**a**) Sparkling wines STAGE 1 (B50, B75, B100), STAGE 2 (S50, S75, S100), STAGE 3 (A50, A75, A100) (**b**) Sparkling wines from STAGE 3 (A50, A75, A100). B50, B75, and B100 (wines before second fermentation with the first addition of bentonite); S50, S75, and S100 (sparkling wines after second fermentation with the second addition of bentonite); A50, A75, and A100 (sparkling wines after 9 months of aging on lees).

Two different PCAs including all the volatile compounds, and the total sum of every group were performed as shown in Figure 5 (40 variables). One PCA (Figure 5a) comprised all the sparkling wine samples from stage 1 (B50, B75, and B100), stage 2 (S50, S75, and S100), and stage 3 (A50, A75, and A100). The analysis determined five principal components (PCs) which explained 90.9% of the total variance, with PC1 (Component 1) and PC2 (Component 2) accounting for 72.4% of the cumulative variance and permitting a significant separation of the samples. Thus, PC1 seemed to explain the effect of the 9 months of aging, discriminating among samples of stages 1 and 2 and those of stage 3. This indicated that the addition of bentonite to base wines did not allow differentiation among treatments and that the different dosages added did not cause major changes, even after the second fermentation. The samples of base wines from stage 1 and sparkling wines from stage 2 were mixed in the right side of the plane over the PC1 axis and the sparkling wines aged 9 months were located on the left side of PC1. Hence, typical aging markers such as diethyl succinate and ethyl lactate were placed on the left side joined to terpenes, which typically increase in concentration during aging due to acid hydrolysis of the glycosidic aroma precursors (Figure 5a). PC2 allowed the separation of the samples after 9 months of aging, depending on the bentonite treatment applied, showing that the fining agent added in the tirage caused greater effects among treatments than the addition of bentonite in the base wine. Figure 5b presents the PCA with only sparkling wines at stage 3, indicating that the first two PCs explained 87.6% of the variance. In this case, the sparkling wines A50 and A75 were grouped on the left side of the plane, and the sparkling wines A100 were grouped on the right side. The corresponding loading distribution clearly reflects the higher enrichment of volatile compounds of the sparkling wines with less quantity of bentonite added during the tirage (Figure 5b), which is in agreement with the results of the chemical and sensorial analyses (Table 1, Figure 2).

## 4. Conclusions

The results of this study showed that the distribution of the dosage of bentonite needed for stabilization of the base wine before and the tirage significantly influences the volatile compounds profile and sensory perception of the sparkling wines. Our results suggest that the amount of bentonite added as a fining agent in the tirage causes greater effects during treatments than the addition of this agent in the base wine. The addition of 100% of the bentonite dosage to the base wine gives rise to wines with higher amounts of volatile compounds; however, the distribution of 75% of the bentonite before the tirage and 25% during it results in a diminution of higher alcohols contents, enhancing the perceived aromatic intensity. From a sensorial point of view, the addition of 50% of the bentonite dosage during the tirage has a negative effect on the foam and aromatic properties. These results reflect the state of the current procedures applied in most wineries; however, knowledge of the effects of the distribution of the dosage could help winemakers with highly unstable wines ensure protein stabilization (because the volatile profile seems to be mostly unaffected) or even enhance the aromatic intensity and complexity of sparkling wines.

## Figures and Tables

**Table 1:** Concentration of volatile compounds of Chardonnay base and sparkling wines along the production process.

Volatile Compounds	LRI	ID	Stage 0	Stage 1			Stage 2			Stage 3		
			BW	B50	B75	B100	S50	S75	S100	A50	A75	A100
ESTERS												
Ethyl butyrate	1055	A	798 ± 101	863 ± 245 ^b^	282 ± 56 ^a^	322 ± 88 ^a^	541 ± 139 ^a^	895 ± 221 ^a,b^*	1163 ± 80 ^b^,*	701 ± 59 ^a^	689 ± 62 ^a^	666 ± 88 ^a^,*
Isoamyl acetate	1122	A	10879 ± 19	8722 ± 1641 ^b^	4773 ± 1095 ^a^	3336 ± 829 ^a^	6019 ± 1166 ^a^	7311 ± 1187 ^a^,*	7873 ± 1093 ^a^,*	707 ± 12 ^a^,*	3677 ± 215 ^b^	3230 ± 144 ^b^
Ethyl hexanoate	1245	A	950 ± 70	988 ± 123 ^b^	597 ± 122 ^a^	653 ± 143 ^a^	836 ± 182 ^a^	804 ± 62 ^a^	963 ± 196 ^a^	6.47 ± 0.46 ^a^,*	758 ± 57 ^b^	844 ± 25 ^b^
Hexyl acetate	1285	A	608 ± 50	123 ± 21 ^a,b^	110 ± 19 ^a^	161 ± 35 ^b^	27.7 ± 3.8 ^a^,*	58.7 ± 5.5 ^b^,*	58.3 ± 14.2 ^b^,*	0.740 ± 0.062 ^a^	13.6 ± 3.0 ^b^,*	14.2 ± 1.0 ^b^,*
Ethyl lactate	1379	A	4.81 ± 1.40	4.66 ± 0.52 ^a^	4.91 ± 1.20 ^a^	5.13 ± 0.25 ^a^	5.11 ± 0.54 ^a^	5.93 ± 0.12 ^a^	5.41 ± 0.82 ^a^	103 ± 1 ^b^,*	6.11 ± 1.05 ^a^	10.1 ± 2.2 ^a^
Ethyl octanoate	1437	A	2440 ± 103	3166 ± 417 ^a^	2773 ± 146 ^a^	2596 ± 81 ^a^	2426 ± 419 ^a^	2449 ± 166 ^a^	2716 ± 397 ^a^	62 ± 23 ^a^,*	1516 ± 80 ^b^,*	1593 ± 37 ^b^,*
Isoamyl hexanoate	1468	A	3.73 ± 0.05	7.71 ± 0.58 ^b^	6.06 ± 1.11 ^a,b^	5.65 ± 0.92 ^a^	4.12 ± 0.56 ^a^,*	5.22 ± 0.89 ^a^	5.72 ± 1.73 ^a^	5.03 ± 1.81 ^b^	2.01 ± 0.09 ^a^,*	2.30 ± 0.10 ^a^,*
Ethyl nonanoate	1558	A	25.6 ± 0.7	43.9 ± 1.6 ^b^	35.4 ± 3.7 ^a^	32.1 ± 0.7 ^a^	35.3 ± 9.9 ^a^	36.7 ± 4.5 ^a^	38.7 ± 8.0 ^a^	49.9 ± 12.8 ^b^	4.51 ± 1.07 ^a^,*	6.54 ± 0.40 ^a^,*
Methyl decanoate	1600	A	13.8 ± 2.9	12.1 ± 1.5 ^a^	12.8 ± 0.6 ^a^	12.2 ± 2.4 ^a^	11.3 ± 2.2 ^a^	12.0 ± 0.3 ^a^	14.4 ± 3.8 ^a^	2.04 ± 0.07 ^b^,*	1.29 ± 0.29 ^a^,*	1.58 ± 0.21 ^a,b^*
Ethyl decanoate	1647	A	1548 ± 92	2773 ± 701 ^a^	2200 ± 252 ^a^	2141 ± 264 ^a^	1558 ± 393 ^a^	1608 ± 249 ^a^,*	1866 ± 485 ^a^	862 ± 74 ^b^	322 ± 54 ^a^,*	418 ± 18 ^a^,*
Isoamy loctanoate	1680	A	114 ± 31	205 ± 53 ^b^	146 ± 9 ^a,b^	117 ± 20 ^a^	123 ± 19 ^a^	93.3 ± 9.7 ^a^,*	137 ± 42 ^a^	6.15 ± 0.57 ^a^,*	13.5 ± 2.8 ^b^,*	16.2 ± 2.7 ^b^,*
Diethy lsuccinate	1675	A	11.5 ± 0.5	14.8 ± 2.6 ^a^	15.4 ± 3.2 ^a^	14.5 ± 3.7 ^a^	15.1 ± 1.9 ^a^	14.1 ± 1.9 ^a^	15.7 ± 2.7 ^a^	nd ^a^,*	36.6 ± 3.2 ^b^,*	43.5 ± 2.0 ^c^*
Ethyl 9-decenoate	1698	B	11.4 ± 0.2	16.9 ± 3.5 ^a^	15.0 ± 1.2 ^a^	14.2 ± 0.6 ^a^	12.6 ± 1.8 ^a^	12.9 ± 0.8 ^a^	14.5 ± 1.6 ^a^	2.83 ± 0.37 ^a^,*	12.1 ± 0.9 ^b^	19.7 ± 1.4 ^c^*
Propyl decanoate	1725	B	4.88 ± 0.09	7.11 ± 1.22 ^a^	5.98 ± 1.03 ^a^	6.10 ± 0.67 ^a^	4.52 ± 0.77 ^a^	4.77 ± 0.28 ^a^	5.53 ± 0.96 ^a^	5.85 ± 0.73 ^b^	0.231 ± 0.031 ^a^,*	0.502 ± 0.031 ^a^,*
Methyl dodecanoate	1823	B	10.7 ± 2.4	4.55 ± 1.03 ^a^	6.57 ± 1.05 ^a,b^	8.51 ± 1.82 ^b^	7.39 ± 1.79 ^a^	7.63 ± 1.34 ^a^	8.90 ± 1.24 ^a^	2.06 ± 0.09 c*	0.673 ± 0.091 ^a^,*	0.903 ± 0.032 ^b^,*
β-phenethyl acetate	1851	A	146 ± 7	108 ± 10 ^a^	120 ± 7 ^a^	120 ± 12 ^a^	78.4 ± 7.0 ^a^,*	89.3 ± 9.6 ^a^,*	95.2 ± 10.5 ^a^,*	1.91 ± 0.11 ^a^,*	21.7 ± 1.1 ^b^,*	38.9 ± 1.5 ^c^*
Ethyl dodecanoate	1864	B	769 ± 30	773 ± 72 ^a^	658 ± 100 ^a^	679 ± 65 ^a^	549 ± 108 ^a^	577 ± 86 ^a^	733 ± 111 ^a^	3.62 ± 1.20 ^a^,*	52.3 ± 6.8 ^b^,*	95.2 ± 6.4 ^c^*
Isoamyl decanoate	1888	A	10.2 ± 1.2	12.8 ± 2.4 ^a^	10.4 ± 2.1 ^a^	11.3 ± 1.6 ^a^	8.33 ± 2.71 ^a^	9.40 ± 1.33 ^a^	14.7 ± 2.7 ^b^	102 ± 10 ^b^,*	0.396 ± 0.015 ^a^,*	1.03 ± 0.18 ^a^,*
Ethyl tetradecanoate	2041	B	37.0 ± 8.7	37.6 ± 10.4 ^a^	34.2 ± 4.3 ^a^	25.1 ± 8.6 ^a^	29.7 ± 6.9 ^a^	31.6 ± 6.2 ^a^	32.8 ± 6.0 ^a^	18.3 ± 4.1 ^b^	7.26 ± 0.32 ^a^,*	19.8 ± 1.6 ^b^
Ethyl hexadecanoate	2235	B	29.6 ± 7.5	37.3 ± 10.8 ^a^	28.1 ± 7.4 ^a^	23.3 ± 4.6 ^a^	27.0 ± 7.5 ^a^	27.2 ± 7.3 ^a^	36.0 ± 2.0 ^a^,*	10.6 ± 3.6 ^b^	4.26 ± 0.60 ^a^,*	12.7 ± 0.9 ^b^,*
ALCOHOLS												
Isobutanol	1074	A	23,739 ± 2359	34,191 ± 2111 ^a^	35,845 ± 14 ^a^	33,422 ± 5141 ^a^	35,867 ± 2617 ^a^	30,621 ± 5268 ^a^	36,070 ± 4798 ^a^	27,497 ± 639 ^a^,*	22,265 ± 3510 ^a^,*	27,906 ± 6129 ^a^
Isoamyl alcohol	1200	A	118,133 ± 865	125,616 ± 10,888 ^a^	121,876 ± 9627 ^a^	124,744 ± 4829 ^a^	120,297 ± 13,684 ^a^	119,670 ± 11,248 ^a^	131,238 ± 19,875 ^a^	122,046 ± 2687 ^b^	96,350 ± 5931 ^a^,*	127,663 ± 11,937 ^b^
Hexanol	1375	A	6115 ± 172	7088 ± 435 ^a^	7113 ± 1177 ^a^	7044 ± 1239 ^a^	7584 ± 1221 ^a^	7176 ± 952 ^a^	8037 ± 1607 ^a^	3612 ± 118 ^a^,*	4688 ± 1077 ^a,b^	6511 ± 232 ^b^
E-3-Hexenol	1366	A	9002 ± 41	9693 ± 150 ^a^	9558 ± 964 ^a^	9756 ± 1126 ^a^	10,312 ± 1212 ^a^	10,184 ± 485 ^a^	11,116 ± 1881 ^a^	7607 ± 2026 ^a^	7318 ± 1286 ^a^	9784 ± 522 ^a^
Phenylethyl alcohol	1940	A	24,449 ± 181	26,394 ± 612 ^a^	26,299 ± 3228 ^a^	25,880 ± 4477 ^a^	26,412 ± 2834 ^a^	24,905 ± 3354 ^a^	28,541 ± 4636 ^a^	19,282 ± 4706 ^a^,*	16,019 ± 996 ^a^,*	28,327 ± 2418 ^b^
TERPENES												
Linalool	1555	A	5.86 ± 0.61	6.01 ± 0.58 ^a^	6.01 ± 0.79 ^a^	5.87 ± 1.32 ^a^	6.17 ± 0.51 ^a^	5.84 ± 0.57 ^a^	6.61 ± 0.94 ^a^	3.16 ± 0.08 ^a^,*	3.55 ± 0.30 ^a^,*	3.74 ± 0.17 ^a^,*
α-terpineol	1693	A	4.20 ± 0.20	3.11 ± 0.77 ^a^	3.17 ± 0.75 ^a^	3.49 ± 1.10 ^a^	3.80 ± 0.55 ^a^	3.74 ± 0.45 ^a^	4.19 ± 1.13 ^a^	3.80 ± 1.11 ^a^	4.25 ± 0.55 ^a^	6.49 ± 0.40 ^b^
Citronellol	1785	A	3.37 ± 0.48	3.19 ± 0.14 ^a^	3.23 ± 0.07 ^a^	3.41 ± 0.66 ^a^	3.01 ± 0.30 ^a^	2.91 ± 0.36 ^a^	3.42 ± 0.43 ^a^	8.82 ± 2.98 ^a^,*	7.47 ± 0.40 ^a^,*	13.0 ± 0.3 ^b^,*
E-nerolidol	2056	A	10.1 ± 0.6	8.70 ± 1.75 ^a^	8.54 ± 0.72 ^a^	9.13 ± 0.69 ^a^	9.12 ± 1.59 ^a^	8.44 ± 0.53 ^a^	9.82 ± 0.90 ^a^	7.90 ± 1.34 ^a^	8.53 ± 0.73 ^a^	14.4 ± 1.1 ^b^,*
ACIDS												
Hexanoic acid	1880	A	10,775 ± 196	10,428 ± 337 ^a^	11,431 ± 1808 ^a^	11,225 ± 2013 ^a^	11,777 ± 1122 ^a^	11,158 ± 1593 ^a^	12,604 ± 1792 ^a^	6053 ± 350 ^a^,*	5132 ± 275 ^a^,*	10,959 ± 797 ^b^
Octanoic acid	2076	A	12,124 ± 220	11,470 ± 655 ^a^	11,689 ± 1355 ^a^	11,071 ± 1642 ^a^	11,839 ± 1702 ^a^	11,387 ± 1532 ^a^	13,122 ± 2352 ^a^	5481 ± 761 ^a^,*	6533 ± 996 ^a^,*	9236 ± 407 ^b^
Decanoic acid	2339	A	4889 ± 25	3620 ± 587 ^a^	3696 ± 376 ^a^	3708 ± 619 ^a^	3414 ± 369 ^a^	3375 ± 491 ^a^	4474 ± 741 ^a^	1040 ± 112 ^a^,*	1190 ± 41 ^a^,*	1972 ± 77 ^b^,*
NORISOPRENOIDS												
Vitispirane a	1518	B	4.53 ± 0.40	2.13 ± 0.32 ^a^	1.86 ± 0.21 ^a^	1.81 ± 0.01 ^a^	2.21 ± 0.31 ^a^	2.59 ± 0.05 ^b^	3.14 ± 0.42 ^b^	9.93 ± 0.81 ^a^,*	8.73 ± 2.15 ^a^,*	8.56 ± 0.46 ^a^,*
Vitispirane b	1522	B	3.53 ± 0.82	1.50 ± 0.01 ^a^	1.65 ± 0.10 ^a^	1.44 ± 0.24 ^a^	1.50 ± 0.43 ^a^	1.93 ± 0.42 ^a^	2.27 ± 0.56 ^a^	6.62 ± 0.31 ^a^,*	6.88 ± 1.79 ^a^,*	5.77 ± 0.11 ^a^,*
TDN	1745	B	12.2 ± 0.1	5.69 ± 1.44 ^a^	4.53 ± 0.59 ^a^	4.82 ± 0.46 ^a^	4.69 ± 0.24 ^a^	6.04 ± 0.12 ^a,b^	7.43 ± 0.45 ^b^	10.9 ± 2.6 ^a^	12.4 ± 1.1 ^a^,*	16.2 ± 1.44 ^b^,*

BW (base wine); B50, B75, B100 (wines before second fermentation with the first addition of bentonite); S50, S75, S100 (sparkling wines after second fermentation with the second addition of bentonite); A50, A75, A100 (sparkling wines after 9 months of aging on lees). Values are expressed in µg/L except the norisoprenoids group which is expressed in relative area × 100. Values with different superscript letters indicate statistically significant differences among the samples of the same stage (*p* < 0.05) by Tukey test *: statistically significant difference with the same sample of the previous stage (*p* < 0.05) by Tukey test. nd: not detected; LRI: linear retention index; ID: reliability of identification: A: mass spectrum and LRI agreed with standards; B, mass spectrum agreed with mass spectral database and LRI agreed with the literature data (Pherobase: www.pherobase.com; NIST Mass Spectrometry Data Center: https://webbook.nist.gov/ (accessed on 20 November 2020)).

## Data Availability

Not applicable.
